# Comparison of Methods of Treating Wounds

**Published:** 1917-11

**Authors:** 


**Comparison of Methods of Treating Wounds.** By Kenneth Taylor, Director, the Robert Walton Goelet Research Fund, American Red Cross Hospital, Paris. Abstracted from the *Journal of the American Medical Association*, Aug. 4, 1917.

T. notes the need of more careful comparison of methods employed in treating wounds, especially infectious ones, and urges systematic standardizing. Often the personal characteristics of the physician are responsible for the superior results obtained from a given method. Even when the same doctor tests two different treatments one after the other, the experience gained in the first may account for better results obtained by the second. The varying vitality of patients must be considered even in military surgery, which deals with men selected for sound health. The age of the wound and the effect of previous treatment must be carefully considered.

Cases to be compared should be grouped according to the *number of days since the injury*. Similarity in the appearance of wounds is frequently misleading, because wounds that seem alike often vary widely by reason of location, vascularity, degree of trauma, — especially microscopic destruction of soft tissues — the presence of foreign matter, and the kind and degree of infection.

The author suggests the following methods of standardization :

1. The selection of cases with essential characteristics in common, which may then be divided into groups for testing various treatments.

2. A slower but logical method is the comparison of large groups of unselected cases. The beds of a service may be divided into two or more groups, and patients assigned upon entrance in rotation. This system must be continued for a long period of time to obtain reliable results.

3. *The most satisfactory method*, the author believes, is the simultaneous treatment of two or more wounds on the same person by the same surgeon.

In judging the efficacy of treatment, T. insists upon attention paid to the following points :

1. Time required for cure, influenced by systemic reaction and complications. A chart should record periodic examinations noting the rate of growth of granulations and epithelium, the disappearance of slough, and evidence of bone repair. Thus an intimate association of treatment and reaction is possible.

2. Measurements of wound surface and observation of the type of scar.

3. Damage to tissues due to the treatment often indicated by painful dressings.

4. Fever reaction due to absorption of toxins; the general comfort of the patient.

5. Extension of infection, retention of purulent discharges because of improper drainage, local cellutis, erysipelas, pyemia, bacteremia, and secondary hemorrhage, often indicate improper treatment.

6. Alteration in bacterial flora compared with the natural alteration during the repair of the wound. a. Disappearance of special bacteria from a suppurating wound due to specificity of antiseptic dressing. b. Frequency of reinfection by a single organism (especially the Bacillus pyocyaneus).

7. The nature of discharges calls for special attention. Suppression of suppuration may be due to failure to react to the infection and may therefore give a bad prognosis.

The process observed when a hypochlorite solution is used, — the dissolving and decolorizing of the solid constituents of a discharge, — may often be deceptive. Although the character of the pus seen on the dressings may change, the activity of the bacteria may remain the same within the granulations and the leukocytes emigrate at the same rate from the infected walls. After such solutions have been withheld for twenty-four hours, the reappearance of the pus cells should not be considered evidence of reinfection.

Only by careful experimentation and observation of this nature can the present confusion in the treatment of infected wounds be avoided and dressings applied efficaciously in particular cases.

